# Supplementary material for: Content-rich biological network constructed by mining PubMed abstracts
Source: BMC Bioinformatics. 2004 Oct 8;5:147. doi: 10.1186/1471-2105-5-147 (PMC528731; doi:10.1186/1471-2105-5-147)
Supplement: Additional File 2 — The original results of the above study (non-essential files are deleted to keep the file size under the limit set by BMC bioinformatics). [file 1471-2105-5-147-S2.bz2 › chilibotAdditionalFile2/dip20/747ID9150216E18129/html/EXBB_TONB.html]

 


 **EXBB** and **TONB** 
  
Found 83 abstracts in PubMed, retrieved 20.  
 

 What does Google say? 
 PDF only 
| .edu only 

---

**Interactive relationship** (e.g. stimulation, inhibition, etc)

**Neutral relationship**- The amino terminal signal anchor of  **TonB**  and its interaction with the cytoplasmic membrane protein  **ExbB**  are essential to this process.  Ref: 11087740 J Biol Chem, 2001
- **ExbB**  and ExbD proteins are part of the  **TonB**  dependent energy transduction system and are encoded by the exb operon in Escherichia coli.  Ref: 12193634 J Bacteriol, 2002
- Both  **ExbB**  and ExbD were also required for association of  **TonB**  with the cytoplasmic membrane.  Ref: 12193634 J Bacteriol, 2002
- Conserved residues Ser 16 and His 20 and their relative positioning are essential for  **TonB**  activity, cross linking of  **TonB**  with  **ExbB** , and the ability of  **TonB**  to respond to proton motive force.  Ref: 11087740 J Biol Chem, 2001
- Deletion of Trp 11 resulted in a  **TonB**  that remained active yet was unable to cross link with  **ExbB** .  Ref: 11087740 J Biol Chem, 2001
- Specifically, both  **ExbB**  and ExbD were found to be required for  **TonB**  to conformationally respond to proton motive force.  Ref: 12193634 J Bacteriol, 2002
- The aim of this review is to describe an in vivo assay of the interactions taking place in the Tol Pal or  **TonB**   **ExbB**  ExbD envelope complexes in the periplasm of Escherichia coli and between them and colicins or g3p protein of filamentous bacteriophages.  Ref: 12423784 Biochimie< MedlineTA>Biochimie, 2002
- Because Trp 11 was demonstrably not involved in the actual cross linking, these results suggest that the  **TonB**   **ExbB**  interaction detected by cross linking occurred at a step in the energy transduction cycle distinct from the coupling of  **TonB**  to the electrochemical gradient.  Ref: 11087740 J Biol Chem, 2001

**Non-interactive relationship** (e.g. studied together, co-existance, homology, etc.)

- However, inducible levels of TX 100 resistance in gonococci had an absolute requirement for the  **TonB**   **ExbB**  ExbD system, suggesting that such resistance in gonococci has an energy requirement above and beyond that required for constitutive pump activity.  Ref: 11796379 Antimicrob Agents Chemother, 2002
- **TonB** , the energy transducer, appears to go through a cycle during energy transduction, with the absence of both  **ExbB**  and ExbD creating blocks at two points i in the inability of  **TonB**  to respond to the cytoplasmic membrane proton motive force and ii in the conversion of  **TonB**  from a high affinity outer membrane association to a high affinity cytoplasmic membrane association.  Ref: 12193634 J Bacteriol, 2002
- Reverse transcription RT PCR, using RNA from organisms grown under iron replete and iron restricted conditions, revealed that  **tonB** ,  **exbB** , exbD, tbpB and tbpA are transcribed as a single unit with expression being up regulated in response to iron restriction.  Ref: 12742718 Vet Microbiol, 2003
- PCR approaches allowed the amplification, cloning and sequencing of A. suis  **tonB** ,  **exbB** , exbD, tbpB and tbpA homologues.  Ref: 12742718 Vet Microbiol, 2003
- In this study, we report the per cell numbers of  **TonB** ,  **ExbB** , ExbD and FepA for cells grown under iron replete and iron limited conditions.  Ref: 11967085 Mol Microbiol, 2002
- The Ton system consisting of the proteins  **TonB** ,  **ExbB**  and ExbD is required for colicin and microcin import.  Ref: 12423780 Biochimie< MedlineTA>Biochimie, 2002
- In Gram negative bacteria, the  **TonB**   **ExbB**  ExbD inner membrane multiprotein complex is required for active transport of diverse molecules through the outer membrane.  Ref: 11737642 Mol Microbiol, 2001
- The ratio of these proteins was calculated at one  **TonB**  two ExbD seven  **ExbB**  under all four growth conditions tested.  Ref: 11967085 Mol Microbiol, 2002
- They share homology with the  **ExbB** , ExbD, and  **TonB**  proteins, respectively.  Ref: 11418549 J Bacteriol, 2001
- Results of insertional mutations, DNA and protein sequence analyses, phage sensitivity tests, transfection tests, complementation tests, and phage adsorption assays together with the cellular location of the proteins indicate that  **TonB** ,  **ExbB** , and ExbD1 are essential for penetration of phage phi L7.  Ref: 12646254 Biochem Biophys Res Commun, 2003
- Both full length  **TonB**  and truncated  **TonB**  consisting of the amino terminal two thirds achieved identical conformations in the cytoplasmic membrane, as determined by their abilities to cross link to the cytoplasmic membrane protein  **ExbB**  and their abilities to respond conformationally to the presence or absence of proton motive force.  Ref: 12823822 Mol Microbiol, 2003
- The energy is transduced to FecA by the inner membrane complex,  **TonB** ,  **ExbB** , and ExbD.  Ref: 12618451 J Bacteriol, 2003
- The reconstitution in Escherichia coli of the S. marcescens Has system demonstrated that haem uptake is dependent on the E. coli  **ExbB** , ExbD and  **TonB**  proteins.  Ref: 11737642 Mol Microbiol, 2001
- , Triton X 100 TX 100 did not require the  **TonB** ,  **ExbB** , or ExbD protein.  Ref: 11796379 Antimicrob Agents Chemother, 2002
- The energy of the proton motive force is postulated to be transduced to the transport proteins by a protein complex that consists of the  **TonB** ,  **ExbB** , and ExbD proteins.  Ref: 11566987 J Bacteriol, 2001
- A complex of three membrane spanning proteins  **TonB** ,  **ExbB**  and ExbD couples the chemiosmotic potential of the cytoplasmic membrane with siderophore uptake across the outer membrane.  Ref: 12409204 Biochim Biophys Acta, 2002
- This gene is clustered with three other genes,  **tonB**   **exbB**  exbD1 exbD2.  Ref: 12646254 Biochem Biophys Res Commun, 2003
- Uptake of colicin Js into sensitive bacteria was dependent on the  **ExbB**  protein but not on the E. coli K 12  **TonB**  and TolA, B, and Q proteins.  Ref: 11395459 J Bacteriol, 2001
- Inducible, but not constitutive, resistance of gonococci to hydrophobic agents due to the MtrC MtrD MtrE efflux pump requires  **TonB**   **ExbB**  ExbD proteins.  Ref: 11796379 Antimicrob Agents Chemother, 2002
- Other biochemical evidence also shows that the proteins that provide energy for iron transport at the outer membrane, such as the  **TonB**   **ExbB**  ExbD system, are structurally very similar across bacterial species.  Ref: 11895294 Curr Top Med Chem, 2001
- **ExbB**  and ExbD do not function independently in  **TonB**  dependent energy transduction.  Ref: 12193634 J Bacteriol, 2002
- Analysis of sequence alignments of TolQ and TolR homologues from numerous Gram negative bacterial genomes, together with analyses of the different tolQ tolR mutants, revealed that the TM domains of TolQ and TolR present structural and functional homologies not only to  **ExbB**  and ExbD of the  **TonB**  system but also with MotA and MotB of the flagellar motor.  Ref: 11722743 Mol Microbiol, 2001
- Quantification of known components of the Escherichia coli  **TonB**  energy transduction system  **TonB** ,  **ExbB** , ExbD and FepA.  Ref: 11967085 Mol Microbiol, 2002
- In gram negative organisms, high affinity transport of iron substrates requires energy transduction to specific outer membrane receptors by the  **TonB**   **ExbB**  ExbD complex.  Ref: 12562789 J Bacteriol, 2003
- Pasteurella multocida  **exbB** , exbD and  **tonB**  genes are physically linked but independently transcribed.  Ref: 12044675 FEMS Microbiol Lett, 2002
- The  **exbB** , exbD and  **tonB**  genes of the Pasteurella multocida animal pathogen have been cloned by complementation of an Escherichia coli  **tonB**  mutant.  Ref: 12044675 FEMS Microbiol Lett, 2002
- Crystal structures of two outer membrane transport proteins, FhuA and FepA, and biochemical data reveal strong long range conformational changes of the proteins upon binding of Fe 3 siderophore complexes and in response to energy transfer from the cytoplasmic membrane into the outer membrane via the  **TonB**   **ExbB**  ExbD protein complex.  Ref: 11934617 Curr Opin Microbiol, 2002
